# Immune Reconstitution Inflammatory Syndrome Unmasking or Worsening AIDS-Related Progressive Multifocal Leukoencephalopathy: A Literature Review

**DOI:** 10.3389/fimmu.2017.00577

**Published:** 2017-05-23

**Authors:** Anna Fournier, Guillaume Martin-Blondel, Emmanuèle Lechapt-Zalcman, Julia Dina, Apolline Kazemi, Renaud Verdon, Emmanuel Mortier, Arnaud de La Blanchardière

**Affiliations:** ^1^Department of Infectious and Tropical Diseases, CHU Côte de Nacre, Caen, France; ^2^Department of Infectious and Tropical Diseases, CHU Toulouse, Toulouse, France; ^3^INSERM U1043 – CNRS UMR 5282, Université Toulouse III, Centre de Physiopathologie Toulouse-Purpan, Toulouse, France; ^4^Department of Pathology, CHU Côte de Nacre, Caen, France; ^5^Department of Virology, CHU Côte de Nacre, Caen, France; ^6^Department of Radiology, CHU Côte de Nacre, Caen, France; ^7^Department of Internal Medicine, Hôpital Louis Mourier, Colombes, France

**Keywords:** immune reconstitution inflammatory syndrome, progressive multifocal leukoencephalopathy, acquired immunodeficiency syndrome, HIV infection, John Cunningham virus, combined antiretroviral therapy, mortality, corticosteroids

## Abstract

Incidence of progressive multifocal leukoencephalopathy (PML) in HIV-infected patients has declined in the combined antiretroviral therapy (cART) era although a growing number of acquired immunodeficiency syndrome (AIDS)-related PML-immune reconstitution inflammatory syndromes (PML-IRIS) have been published during the same period. Therapeutic management of PML-IRIS is not consensual and mainly relies on corticosteroids. Our main aim was, in addition to provide a thoughtful analysis of published PML-IRIS cases, to assess the benefit of corticosteroids in the management of PML-IRIS, focusing on confirmed cases. We performed a literature review of the 46 confirmed cases of PML-IRIS cases occurring in HIV-infected patients from 1998 to September 2016 (21 unmasking and 25 paradoxical PML-IRIS). AIDS-related PML-IRIS patients were mostly men (sex ratio 4/1) with a median age of 40.5 years (range 12–66). Median CD4 T cell count before cART and at PML-IRIS onset was 45/μl (0–301) and 101/μl (20–610), respectively. After cART initiation, PML-IRIS occurred within a median timescale of 38 days (18–120). Clinical signs were motor deficits (69%), speech disorders (36%), cognitive disorders (33%), cerebellar ataxia (28%), and visual disturbances (23%). Brain MRI revealed hyperintense areas on T2-weighted sequences and FLAIR images (76%) and suggestive contrast enhancement (87%). PCR for John Cunningham virus (JCV) in cerebrospinal fluid (CSF) was positive in only 84% of cases; however, when performed, brain biopsy confirmed diagnosis of PML in 90% of cases and demonstrated histological signs of IRIS in 95% of cases. Clinical worsening related to PML-IRIS and leading to death was observed in 28% of cases. Corticosteroids were prescribed in 63% of cases and maraviroc in one case. Statistical analysis failed to demonstrate significant benefit from steroid treatment, despite spectacular improvement in certain cases. Diagnosis of PML-IRIS should be considered in HIV-infected patients with worsening neurological symptoms after initiation or resumption of effective cART, independently of CD4 cell count prior to cART. If PCR for JCV is negative in CSF, brain biopsy should be discussed. Only large multicentric randomized trials could potentially demonstrate the possible efficacy of corticosteroids and/or CCR5 antagonists in the management of PML-IRIS.

## Introduction

Progressive multifocal leukoencephalopathy (PML), first described by Astrom in 1958 and finally related to the John Cunningham virus (JCV) by Padgett in 1971, is a rare devastating disease of the CNS caused by the reactivation of JCV in immunocompromised patients (1). PML results in death and loss of the infected oligodendrocytes and their myelin sheaths, leading to a demyelinating disease with bizarre astrocytes and enlarged oligodendrocyte nuclei (2, 3). Before the HIV epidemic, PML was exclusively observed in patients with hematological malignancies, organ transplant recipients, or chronic inflammatory disorders with immunosuppressive drugs. Although HIV infection currently accounts for approximately 80% of new PML cases, PML occurrence related to the use of therapeutic monoclonal antibodies including natalizumab, efalizumab, and rituximab is rising (4). No drug is effective against JCV. Since the restoration of CD4 and CD8 JCV-specific T cell immune responses, allowing the control of JCV replication, the initiation of combined antiretroviral therapy (cART) in HIV-infected patients or the discontinuation of immunosuppressive drugs in non-HIV-infected patients remain the only available therapeutic alternative for PML. Indeed, cART-induced immune recovery improved PML survival in HIV-infected patients from 0–30 to 38–62% after 1 year (1, 5). However, immune restoration is not always beneficial, and 16.7% of HIV-infected patients with PML worsen after cART initiation, due to severe neuroinflammation within settings of immune reconstitution inflammatory syndrome (PML-IRIS) (6). Pathophysiology of PML-IRIS implicates a distorted cART-induced immune restoration of JCV-specific immune responses dominated by cytotoxic CD8 T cells, generating inflammatory CNS tissue damage, neurological deficits, and pronounced mortality and morbidity (7, 8). The contribution of IRIS toward the clinical worsening of PML is difficult to distinguish from natural evolution of classical AIDS-associated PML. PML-IRIS diagnosis requires a demonstration of HIV replication control on cART—which is a prerequisite to immune recovery—and of an inflammatory response in the infected brain through the detection of gadolinium enhancement and/or edema or mass effect of PML lesions by MRI, and/or parenchymal infiltration of PML lesions by predominantly CD8 T cells and macrophages in brain biopsies (9, 10). Therapeutic management of PML-IRIS usually relies on steroids, despite fears with regard to the use of treatment modalities that may blunt the anti-JCV immune responses that are instrumental in the long-term control of JCV replication (11, 12). The aim of this review was to analyze epidemiological, clinical, radiological, biological, pathological, and therapeutic data on confirmed PML-IRIS published in the literature, to assess the benefit of corticosteroids in the management of PML-IRIS, and to provide incentive for future large-scale studies.

## Methods

A review of the literature in English, French, and Spanish was performed via a computer-based search on PubMed Home by crossing the key words “HIV infection” and “progressive multifocal leukoencephalopathy” with or without “immune reconstitution inflammatory syndrome.” Articles were selected for review if their title or abstract suggested that they either reported individual or group data with PML-IRIS diagnosis or performed a review on PML-IRIS in HIV-infected patients. Searches in the reference lists of selected articles led to the identification of further relevant publications. The literature search period ranged from the first case described in 1998 to September 2016. We took care not to list the same case twice.

Diagnosis of AIDS-related PML-IRIS required three major criteria drawn from PML diagnostic criteria as per the AAN consensus statement and CNS-IRIS criteria [2].

– HIV-infected patient
– Criteria for definite PML:
  ◦ Subacute onset of neurological deficits
  ◦ MRI features
  ◦ Presence of JCV DNA in cerebrospinal fluid (CSF) and/or brain tissue.
– Criteria for definite PML-IRIS in adult HIV-infected patients:
  ◦ Administration of effective cART within the previous 2 years
  ◦ Subacute onset of neurological deficits that either appeared (unmasking-PML-IRIS) or was exacerbated after initiation of cART (paradoxical-PML-IRIS).
  ◦ Decrease in plasmatic HIV RNA viral load (VL) of ≥1 log_10_ copies/ml with or without an increase in CD4 T cell count from baseline at the onset or worsening of neurological symptoms.
  ◦ Evidence of an inflammatory reaction in the brain demonstrated by MRI (contrast enhancement, edema and/or mass effect) and/or CNS histopathology if available (T cell infiltration), to distinguish PML-IRIS from usual progression of previously acquired PML.

We analyzed the following parameters for each case: Epidemiological characteristics, timescale between cART initiation and PML-IRIS onset, clinical presentation, brain MRI signs (hypointense lesions in T1-weighted and hyperintense in T2-weighted sequences and FLAIR images), contrast enhancement, edema, mass effect, HIV VL and CD4 T cell count at baseline and PML-IRIS diagnosis, results of PCR for JCV in CSF, detection of JCV and pathological data in brain biopsy, clinical outcome and corticosteroid or other therapy if administered. Statistical analysis was performed using STATA 14 to compare the outcome according to drug therapy with or without corticosteroids. Proportions were compared using the Chi-square test or Fisher’s exact test, as appropriate. A p-value of <0.05 was considered to be statistically relevant.

## Results of the Literature Review

### Epidemiology

Through our literature review from 1998 to September 2016, we identified 157 cases published as having developed “PML-IRIS”. Among them, 113 cases were excluded because they did not satisfy the required definite PML-IRIS criteria. We finally identified 46 confirmed and well-described cases of PML-IRIS in HIV-infected patients (9–11, 13–39; Fournier et al., under review^1^). All cases were virologically confirmed-PML. Twenty one (46%) cases were unmasking PML-IRIS and 25 (54%) were paradoxical PML-IRIS. Patient characteristics are summarized in Tables 1 and 2 (Tables 1 and 2).

**Table 1:** **Epidemiological, clinical, and radiological characteristics of the 46 included patients**.

	Value	*N*
Sex ratio (male/female)	4	42
Median age, years (range)	40.5 [12–66]	38
Median delay between cART and IRIS, days (range)	38 [18–120]	33
Unmasking PML-IRIS	41 [20–120]	14
Paradoxical PML-IRIS	38 [18–120]	19
Clinical features		
Motor deficit (%)	27 (69%)	39
Speech disorders (%)	14 (36%)	39
Cognitive disorders (%)	13 (33%)	39
Cerebellar ataxia (%)	11 (28%)	39
Visual disturbances (%)	9 (23%)	39
Facial paralysis (%)	4 (10%)	39
Seizure (%)	3 (8%)	39
Brain MRI abnormalities		
Hypo intense lesions on T1-weighted and hyper intense lesions on T2-weighted and FLAIR sequences (%)	35 (76%)	46
Contrast-enhanced lesions (%)	40 (87%)	46
Edema (%)	14 (30%)	46
Mass effect (%)	11 (24%)	46

*N: number of data available for characteristic*.

**Table 2:** **Biological, virological, and pathological characteristics of the 46 PML-IRIS patients**.

	Value	*N*
Median HIV viral load (VL) before combined antiretroviral therapy (cART) (/ml) (range)	100,000 [29–1,000,000]	31
Unmasking PML-immune reconstitution inflammatory syndromes (PML-IRIS)	100,000 [40–750,000]	13
Paradoxical PML-IRIS	125,000 [29–1,000,000]	18
Median HIV VL at IRIS onset (/ml) (range)	490 [10–100,000]	30
Unmasking PML-IRIS	100 [40–100,000]	14
Paradoxical PML-IRIS	710 [10–10,000]	16
Median CD4 T cell count before cART (/μl) (range)	45 [0–301]	37
Unmasking PML-IRIS	48 [8–301]	18
Paradoxical PML-IRIS	43 [0–284]	19
Median CD4 T cell count at IRIS onset (/μl) (range)	101 [20–610]	28
Unmasking PML-IRIS	101 [49–464]	10
Paradoxical PML-IRIS	101 [20–610]	18
Positive PCR for JCV in cerebrospinal fluid (%)	32 (84%)	38
Unmasking PML-IRIS	17 (94%)	18
Paradoxical PML-IRIS	15 (75%)	20
Detection of John Cunningham virus in brain biopsy (%)	19 (90%)	21
Pathologically proven PML (%)	10 (48%)	21
Pathologically proven PML-IRIS (%)	20 (95%)	21

*N, number of data available for characteristic*.

### Diagnosis

Median delay between cART initiation and the onset of unmasking and paradoxical PML-IRIS was 41 and 38 days, respectively. PML-IRIS presented mostly with motor deficits, speech disorders, cognitive disorders, cerebellar ataxia, visual disturbances, facial paralysis, and/or seizures (Table 1).

All patients underwent brain MRI that revealed contrast-enhanced lesions, edema, and mass effect in 87, 30, and 24% of cases. Only six patients presented none of these signs (Table 1).

Among the cases for which information was available, median HIV VL before initiating cART was relatively high (100,000 cp/ml), declining rapidly thereafter at PML-IRIS onset to 490 cp/ml, with no difference between the two types of PML-IRIS. Likewise, among cases for which information was available, median blood CD4 T cell count before cART was 45/μl (range 0–301), increasing upon diagnosis of PML-IRIS to 101/μl (range 20–610), with no difference between the two types of PML-IRIS (Table 2).

Among the 38 patients who underwent PCR analysis to determine the presence of JCV in the CSF, PCR was positive in 84% of cases. Among the 21 patients who underwent brain biopsy, 10 (48%) presented one or more histological signs of PML (multifocal demyelination, oligodendrocytes containing inclusion bodies, enlarged bizarre astrocytes with reactive gliosis), and PML was virologically confirmed for 19 (90%): 7 by PCR in brain tissue, 7 by immunohistochemical detection and 5 by *in situ* hybridization (Table 2). When PCR for JCV in CSF and virological studies in brain biopsy were performed simultaneously, presence of JCV was positive in CSF and brain tissue in 5/13 patients (39%), positive in CSF but negative in brain tissue in 2/13 patients (15%), and negative in CSF but positive in brain tissue in 6/13 patients (46%) (Table 2).

Among the 21 patients who underwent brain biopsy, 20 (95%) presented histological evidence of IRIS, including the 6 patients without contrast enhancement, edema or mass effect on brain MRI (Table 2). Histological features of PML-IRIS were the parenchymal accumulation of T cells and activated macrophages in 12/20 biopsies (60%). T cell infiltrates were composed of CD8 T cells for 12/20 biopsies (60%). When specified, CD4 T cells were localized in the perivascular compartment without infiltrating the parenchyma. Cells from the B cell lineage were found in 12/20 cases (60%), including CD20^+^ B cells for 7/20 cases (35%) and CD138^+^ plasmocytes for 5/20 cases (25%). Oligodendrocytes contained JCV inclusion bodies in 8 (40%) cases. Unfortunately, 8/20 (40%) biopsied cases report only a perivascular infiltrate with T cells without specifying their CD4 or CD8 specificity.

### Drug Therapy and Clinical Outcome

Regarding management of PML-IRIS, 29 of the 46 patients (63%) were treated with corticosteroids: methylprednisolone alone in 3 cases (10%), methylprednisolone then prednisone or dexamethasone in 5 cases (17%), prednisone or dexamethasone directly in 14 cases (48%), or an unspecified corticosteroid in 7 cases (24%) with variable or non-specified modalities for dosage and duration. Among the remaining patients, 4 were treated with cidofovir and 1 with maraviroc (Table 3).

**Table 3:** **Treatment and clinical outcomes of the 46 PML-IRIS patients**.

	Value	*N*
Treatment		
Corticosteroid (%)	29 (63%)	46
Cidofovir (%)	4 (9%)	46
Maraviroc (%)	1 (2%)	46
Clinical outcome independent of therapy		
Improvement (%)	27 (63%)	43
Stabilization (%)	4 (9%)	43
Death within 2 years (%)	12 (28%)	43
Clinical outcome according to therapy		
With corticosteroids		
Improvement or stabilization (%)	21 (72%)	29
Aggravation then death (%)	8 (28%)	29
Without corticosteroids		
Improvement or stabilization (%)	10 (71%)	14
Aggravation then death (%)	4 (29%)	14

*N, number of data available for characteristic*.

Among the 43 patients for whom outcome is detailed with a median follow-up of 8 months (0–101 months), 31 patients (72%) improved or stabilized, whereas 12 (28%) worsened then died from PML-IRIS within the 2 years following diagnosis, with no significant difference between unmasking and paradoxical PML-IRIS, respectively, 72 vs 56% improved, 6 vs 12% stabilized and 22 vs 32% died.

Clinical outcome of PML-IRIS did not differ between patients treated or untreated by corticosteroids, whatever be the type of PML-IRIS: 21/29 (72%) of patients who received corticosteroids had a favorable outcome (improvement or stabilization) vs 10/14 (71%) of the patients who did not receive corticosteroids (Table 3).

## Discussion

### Epidemiology

From 1998 to September 2016, we identified in the literature 46 definite cases of HIV-related PML-IRIS. This exhaustive review that aimed to assess the impact of steroids on the outcome of definite AIDS-related PML-IRIS excluded voluntarily cases that could have been considered as unfavorable progression of PML despite cART. We are therefore unable to precise the incidence of PML-IRIS. Because IRIS has only been considered as a distinct clinical condition since the late 1990s, it is likely that many reported cases of PML occurring or worsening after cART initiation were not identified as PML-IRIS. Furthermore, as demonstrated in our review, some cases of PML-IRIS have been histologically documented without brain MRI signs.

If most PML-IRIS patients were severely immunocompromised with a median CD4 cell count before cART of 45/μl, the disease may occur in patients with relatively high CD4 cell count before cART initiation (100–300 CD4/μl). In contrast, the modest increase in blood CD4 T cell count after cART at PML-IRIS onset (101 CD4/μl) is comparable with the mean CD4 T cell count of 84–104^/^μl reported in PML patients before the cART era (2). Meta-regression analysis revealed that a CD4 cell count <50 cells/μl upon cART initiation was an independent risk factor for IRIS, independently of the type of opportunistic infection (9). Likewise, Shelburne et al. reported that predictive factors for *Mycobacterium tuberculosis, Mycobacterium avium* complex, and *Cryptococcus neoformans*-associated IRIS were antiretroviral drug naivety, active or subclinical opportunistic infection with high antigen burden before cART initiation, CD4 count <50 cells/μl and a rapid initial decrease in HIV VL in response to cART (40). Among AIDS-related PML patients, the only identified risk factor for PML-IRIS is a lower baseline CD4 T cell count (mean 34.8 vs 71.7 for PML) (41).

Among PML-IRIS patients, we observed an almost identical number of cases of unmasking and paradoxical PML-IRIS, whereas diagnostic of unmasking is much more difficult to establish. There was no difference in the median delay between cART initiation and diagnosis of PML-IRIS (41 and 38 days, respectively). In their review of 54 PML-IRIS patients (also including non-definite PML-IRIS cases), Tan et al. observed a trend for paradoxical PML-IRIS to develop IRIS sooner compared to unmasking PML-IRIS after cART initiation (4 vs 8 weeks) (11). In our study, we only observed a trend toward a greater attributable mortality for paradoxical PML-IRIS compared to unmasking PML-IRIS (32 vs 22%), as did Tan et al. (53 vs 31% of cases) (11).

### Diagnosis

Strikingly, in our literature review, we had to exclude 113 published cases that failed to fit minimal diagnostic criteria for PML-IRIS and that may have been related to an unfavorable outcome of PML despite cART initiation. Although particularly challenging, differentiating unfavorable outcome of PML from PML-IRIS remains crucial since treatment strategies drastically differ between the two settings. Indeed, PML-IRIS requires therapeutic control of antiviral inflammatory response, whereas PML treatment aims at restoring JCV-specific immune responses to afford viral clearance.

With similar frequencies of motor deficit (69 vs 42%), speech disorders (36 vs 40%), cognitive disorders (33 vs 36%), cerebellar ataxia (28 vs 29%), and visual disturbances (23 vs 19%), the clinical presentation of PML-IRIS patients did not differ from PML patients reported in the pre-cART era, with the exception of subacute onset of PML-IRIS that is very different from the usual progressive onset of PML (42).

Brain MRI pattern of PML-IRIS with multiple subcortical white matter lesions appearing hypointense on T1 sequences and hyperintense on T2 and FLAIR sequences did not differ from PML, except for demonstration of inflammatory features that, although inconstant, remain instrumental clues for PML-IRIS diagnosis (1, 2). Rarely associated with PML in the pre-cART era (5–10%), gadolinium enhancement of PML lesions (87%), peri-lesional edema (30%), and mass effect (24%) were key findings in published cases (43–45). Although 13% of these 46 patients had no brain inflammatory features on brain MRI, PML-IRIS was confirmed by histology. Consequently, while contrast enhancement on brain MRI supports PML-IRIS diagnosis, its absence does not preclude it, and diagnosis of PML-IRIS may require brain biopsy (5, 10).

In contrast with patients with classical PML who present minimal infiltrates predominantly composed of CD68^+^ macrophages/microglial cells with very few T cells, most of the 21 patients with PML-IRIS who underwent brain biopsy displayed a massive accumulation of T cells in PML-IRIS lesions (95%). These inflammatory infiltrates are dominated by CD3^+^ T cells, which are almost exclusively composed of CD8^+^ T cells (1, 2, 5, 24), reminiscent of the pathology described in PML-IRIS occurring in non-HIV-infected patients (46). Although a case report suggested the central role of polyfunctional CD4 T cells in natalizumab-associated PML-IRIS, CD4 T cells do not usually infiltrate the CNS parenchyma in HIV-infected patients experiencing PML-IRIS but remain sequestrated in the perivascular vicinity while CD8 T cells penetrate the parenchyma and interact with JCV-infected oligodendrocytes (10, 47). It remains to be determined whether the infiltrating CD8 T cells are solely directed against JCV or are specific for other (self-) antigens in the context of a dysregulated T-cell response, although physical contact of CD8 T cells expressing Granzyme B in close apposition to JCV-infected oligodendrocytes point toward a role for JCV-specific CD8 T cells.

Although essential for PML-IRIS diagnosis when brain MRI is not contributive, potential complications of brain biopsy nevertheless stress the need for an alternative diagnostic marker.

In such conditions, proton magnetic resonance spectroscopy could be of interest in demonstrating specific modifications. In particular, it was shown that Lipid1/Creatin ratio is elevated in PML-IRIS lesions independently of contrast enhancement, representing a surrogate marker for PML-IRIS, but only myoinositol/creatine remains elevated in lesions of patients with PML-IRIS over time (34). Others authors underlined an additional increase of choline and lactate/lipids in a case report using stereotactic brain biopsy for confirmed PML-IRIS compared with the spectrum of PML acquired before cART (48). The hypoperfusion of brain PML lesions determined by arterial spin labeling MRI was significantly greater in PML progressors than in survivors and is inversely correlated to the occurrence of PML-IRIS in one interesting study that deserves confirmation (49). Finally, although monitoring changes in the numbers or in the functional profile of circulating JCV-specific CD4 and CD8 T cells after initiating cART appeared promising for PML-IRIS diagnosis, PML-IRIS was not consistently associated with a higher frequency of circulating JCV-specific CD4 or CD8 T cells, suggesting that other T cell subsets play a role in the pathogenesis of PML-IRIS (50).

A consensual diagnostic statement for PML-IRIS based on a multiparametric assessment is still lacking, but urgently needed. Here, we propose such a definition, adapted from Tan K et al., which should be validated in further studies (Table 4) (11).

**Table 4:** **Proposed diagnostic criteria for AIDS-related PML-IRIS**.

(i) HIV infection (ii) Close temporal relationship between cART initiation and disease onset (iii) cART resulting in a decrease in plasma HIV VL > 1 log_10_ at the onset or worsening of neurological symptoms (iv) Diagnosis of PML by detection of JCV DNA in CSF or brain tissue (v) Evidence of an inflammatory reaction demonstrated by MRI and/or CNS histopathology (vi) Symptoms could not be explained by a newly acquired infection, the expected course of PML or another opportunistic infection or drug toxicity

### Prognosis and Drug Therapy

Although survival of patients with PML has improved over recent years, thanks to earlier recognition, improvement in diagnostic techniques, and the widespread use of cART, the mortality rate reaches around 50% of HIV-infected patients developing PML (51, 52). In our literature review, we observed a 28% mortality rate of PML patients developing IRIS. Several retrospective observational studies have demonstrated the absence of any significant difference in survival between PML with and without IRIS (53, 54). In the absence of any effective anti-JCV treatment, PML outcomes indeed depend entirely on the individual’s capacity to recover the JCV-specific immune responses that can control viral replication. In HIV-infected patients, cART is the only therapeutic alternative with a proven benefit on PML outcome, with the risk of developing IRIS in a subset of patients as a testimony of the immune system’s ability to control JCV replication and to improve PML outcome (55).

However, PML-IRIS can, in rare cases, become a severe and life-threatening condition while its management using corticosteroids and/or any other immune modulatory therapy remains non-consensual. Steroids have been used and may be effective in IRIS following AIDS-related CNS infections caused by mycobacteria, cryptococci, and cytomegalovirus (41). Corticosteroids have, in certain cases, been successfully used for catastrophic PML-IRIS (massive inflammation resulting in impending brain herniation) at a high and early intravenous dosage and over a long duration, but with no apparent difference in the duration of survival for surviving patients, or time to death for deceased patients (11, 21, 30, 56). On the other side, a potentially deleterious role of corticosteroids on long-term anti-JCV immune responses has been raised (12). Our work that included only definite PML-IRIS patients in order to exclude PML patients potentially misclassified as PML-IRIS does not demonstrate a potential benefit of corticosteroids on the prognosis of HIV-related PML-IRIS, nor a deleterious effect, possibly due to the small number of cases.

Alternative therapeutic strategies that selectively alleviate inflammation without hampering immune restoration are therefore desirable. Recent insights arising from the management of inflammatory forms of PML in IRIS settings suggest the role of the CCR5-CCL5 axis in PML-IRIS pathogenesis (57). A dramatic clinical improvement experienced by an HIV-infected patient developing PML-IRIS—a case which could not be included in our review—was reported when maraviroc, an antagonist of CCR5, was added to ART (58). It was suggested that maraviroc may have reduced the severity of neuroinflammation related to IRIS by targeting CCR5^+^ leukocyte recruitment to the CNS (59). This concept was also strengthened by a telling case report of a woman with multiple sclerosis, who developed natalizumab-associated PML and who benefited from the preventive and curative properties of maraviroc for PML-IRIS. In addition, a selective reduction in the proportions of CCR5^+^ T cells in CSF was observed while the patient was receiving maraviroc, suggesting that CCR5 antagonists may selectively limit immune cell trafficking into the CNS *in vivo* (60). These observations were further developed, showing that the vast majority of CD8^+^ T cells infiltrating PML-IRIS lesions, the likely drivers of tissue damage, express high levels of CCR5 (59, 61). Building on these observations, it was suggested that CCR5 might be implicated in the pathogenesis of PML-IRIS, hence warranting further studies. The single patient in our review who received maraviroc and the very few contradictory published case reports prevent us from drawing conclusions on the potential immunomodulatory benefits of maraviroc in inflammatory PML settings (29, 38, 58, 60). Worthy of note, the double-blinded randomized placebo-controlled CADIRIS trial showed that the addition of maraviroc to cART did not reduce the risk of IRIS in 276 patients with advanced HIV infection (62). However, this study included a heterogeneous cohort of patients with IRIS, with only very few opportunistic infections affecting the CNS, precluding the generalization of its conclusions to IRIS affecting the CNS.

Therefore, in the absence of clinical multicentric randomized trials, no recommendations can be made on the use of corticosteroids and/or maraviroc in the management of PML-IRIS. However, we believe that steroids should be reserved for PML-IRIS patients with severe neurological symptoms and/or massive inflammation with cerebral edema or even brain herniation (11, 21, 30, 56) and not be used when patients only displayed contract-enhanced lesions on brain MRI without a clinical worsening related to this inflammatory reaction. Preclinical studies aimed at delineating the behavior of CCR5^+^ JC-virus-specific T cells in blood and CSF are needed to support clinical trials assessing the impact of CCR5 antagonists in the context of inflammatory PML (57).

## Conclusion

Although classical PML has declined with cART, PML-IRIS now accounts for up to 25% of PML cases diagnosed in HIV-infected patients during the cART era. Diagnosis of PML-IRIS should be considered in any HIV-infected patient developing unexplained neurological disorders or worsening of a previously diagnosed PML 2 weeks to 4 months after the initiation or resumption of effective cART, independently of the CD4 cell count prior to cART. Brain MRI is instrumental by demonstrating inflammatory features of PML lesions and could be complemented by brain biopsy in the absence of contrast-enhanced lesions. Although it can be severe, the outcome of AIDS-related PML does not seem to be worsened by IRIS, which, instead, is a testimony of a protective immune response that is instrumental in the long term of JCV replication. Our current perception of this neuroinflammatory disease supports therapeutic strategies aimed at modulating immune aggression without dampening the life-saving restoration of the immune response. No definite conclusion can be drawn on the efficacy of steroids, although case reports suggest that early corticosteroid therapy may be proposed for life-threatening PML-IRIS. There is an urgent need for randomized multicentric trials using corticosteroids and/or maraviroc to improve prognosis of AIDS-related PML-IRIS. Future studies should also be designed to better delineate pathophysiological mechanisms underlying PML-IRIS and identify plasmatic and/or CSF biomarkers of this difficult to diagnose entity.

## Ethics Statement

Since this study was only a review, it did not require ethical approval.

## Author Contributions

AF and AB designed the study, collected and analyzed data, and drafted the manuscript. GM-B participated in manuscript drafting. AF performed statistical analysis. All authors critically commented on the paper, contributed toward and approved the final manuscript.

## Conflict of Interest Statement

The authors declare that the research was con-ducted in the absence of any commercial or financial relationships that could be construed as a potential conflict of interest.

## Footnotes

^1^Fournier A, Martin-Blondel G, Lechapt-Zalcman E, Dina J, Kazemi A, Verdon R, et al. Two new cases of HIV-related progressive multifocal leukoencephalopathy unmasked or worsened by an immune reconstitution inflammatory syndrome. (Under Review in *Frontiers in Immunology*).
